# A comparison of coastal habitat restoration projects in China and the United States

**DOI:** 10.1038/s41598-019-50930-6

**Published:** 2019-10-07

**Authors:** Shanze Li, Tian Xie, Steven C. Pennings, Yuchun Wang, Christopher Craft, Mingming Hu

**Affiliations:** 1 State Key Laboratory of Simulation and Regulation of Water Cycle in River Basin, China Institute of Water Resources and Hydropower Research, Beijing, 100038 China; 2 Department of Water Environment, China Institute of Water Resources and Hydropower Research, Beijing, 100038 China; 3 School of Environment, Beijing Normal University, State Key Joint Laboratory of Environmental Simulation and Pollution Control, Beijing, 100875 China; 4 Department of Biology and Biochemistry, University of Houston, Houston, TX 77204 USA; 5 School of Public and Environmental Affairs, Indiana University, Bloomington, IN 47405 USA

**Keywords:** Environmental impact, Sustainability

## Abstract

We compared coastal restoration projects in a developing country, China, and a developed country, the United States of America, both of which are facing loss and degradation of coastal habitats at similar latitudes, for the period of 1992–2014. To document the scale of coastal habitat restoration projects in the two countries, we identified 914 coastal restoration projects with an accumulated area of 300,521 acres in China, with most of our information coming from scientific papers, and 1,620 coastal restoration projects with an accumulated area of 243,064 acres in the USA, with most of our information coming from public databases. In both countries, about half the projects were in wetland habitats, but China had a greater proportion of projects in submerged habitats (43% versus 28% in the USA) and the USA a greater proportion in coastal upland habitats (21% versus 9% in China). The number of new projects steadily increased over time in China, but dropped after 2006 in the USA, although the total cost of new projects continued to increase. The number of projects in China and the total cost of projects in the USA were correlated with national GDP. Restoration projects in China used fewer techniques, had fewer partners, and took longer to complete than projects in the USA. Information about projects was incomplete, especially in China, and both countries could do more to make information publically available. We know more about project construction than project outcomes, and it is unclear whether projects are achieving their goals or whether the techniques used are optimal.

## Introduction

Coastal habitats play important roles in providing ecosystem services by supporting important organisms, preventing seawater intrusion, conserving biodiversity, moderating microclimate, and promoting nutrient cycling and carbon sequestration^1–4^. However, coastal regions are at considerable risk from natural disturbances and anthropogenic stresses^5^.

Natural disturbances in coastal ecosystems include hurricanes, storms^6^, saline intrusion, sea level rise^7,8^, fire, outbreaks of herbivores^9,10^ and wrack^11,12^. Sea level rise is one of the greatest threats to coastal habitats around the globe^13–15^ and it can lead to saline intrusion into groundwater, loss of coastal habitat^16^, economic costs to coastal industries, human suffering and increased mortality when sea level rise exacerbates extreme events such as storms^17^.

In addition to sea level rise, regional anthropogenic stresses also threaten coastal habitats^18^. There is a long history of developed and developing countries, including the Netherlands^19^, the United States of America^20^, Japan^21^, Australia^22^, South Korea, North Korea and China^23,24^ seeking to expand and exploit coastal lands to meet the needs of their growing populations. As a result, coastal habitats have been impacted by shoreline armoring, aquaculture, ports, agriculture, urbanization, salt extraction, industrial development, natural resource extraction and dikes^23,25^. For example, over half of the natural coastal habitats in China have been lost during the last 60 years^23,26^.

Such disturbances, whether natural or anthropogenic, threaten coastal habitats not only directly by affecting primary and secondary productivity^6^, community composition and distribution, and biodiversity^23^, but also indirectly, by affecting natural processes by reducing habitat heterogeneity and connectivity^2,23^. Concurrent with the loss of community structure and ecosystem functions is a loss of ecosystem services that degrade the quality of life of human populations.

In an attempt to mitigate for habitat loss, many coastal restoration projects have been conducted^27–31^. Globally, wetland restoration is driven by policies such as the Ramsar convention on wetlands of international importance, the Clean Water Act of the US, the Water Framework Directive of the European Union, and others^30^. The earliest restoration efforts that we are aware of involved planting mangroves for fuel and wood. Large-scale mangrove afforestation efforts date back to the end of 19^th^ century or earlier in Indochina^32^. About 100 years ago, salt marsh vegetation was planted in the USA, Western Europe, Australia, and New Zealand to protect coastal habitats from erosion^33–35^. Around the same time, degraded freshwater wetlands were first irrigated with freshwater to rebuild waterfowl habitat^36^. These early restoration activities focused on restoring a particular function such as wood production, shoreline protection, or waterfowl production. Restoration today consists of reestablishing a variety of ecological attributes including community structure (species diversity and habitat) and ecosystem processes (energy flow and nutrient cycling), and as a result a broad spectrum of goods and services delivered by healthy, functioning wetlands^30^.

Whereas the history of coastal restoration is relatively long, success and failure coexist. To document the history and scale of coastal restoration projects, and to evaluate approaches to date, we selected the People’s Republic of China (henceforth China) and the United States of America (henceforth USA), to compare a developing country and a developed country. China and the USA are large countries of roughly the same size and shape: the coastline of China is 18,436 km (coastline source from State Oceanic Administration People’s Republic Of China at www.soa.gov.cn) and the area of the country is 9,569,901 km^2^, giving a coast:area ratio of 1.90; the coastline of the USA is 12,383 km (coastline source from Department of Commerce, National Oceanic and Atmospheric Administration, National Ocean Service at http://www.noaa.gov/) and the area of the country is 9,161,966 km^2^ (The World Factbook 2014–15), giving a coast:area ratio of 1.35. We reviewed coastal restoration projects conducted in these two countries between 1992 and 2014 in order to evaluate similarities and differences in the restoration projects, including the types of habitats restored, the historical pattern of restoration, the number and size of projects, the funding sources for projects, the number and type of partners involved, and how information about projects is made available to the public. We discuss possible explanations for the patterns found, and suggest some improvements to future practices.

## Results

**Figure 1:**
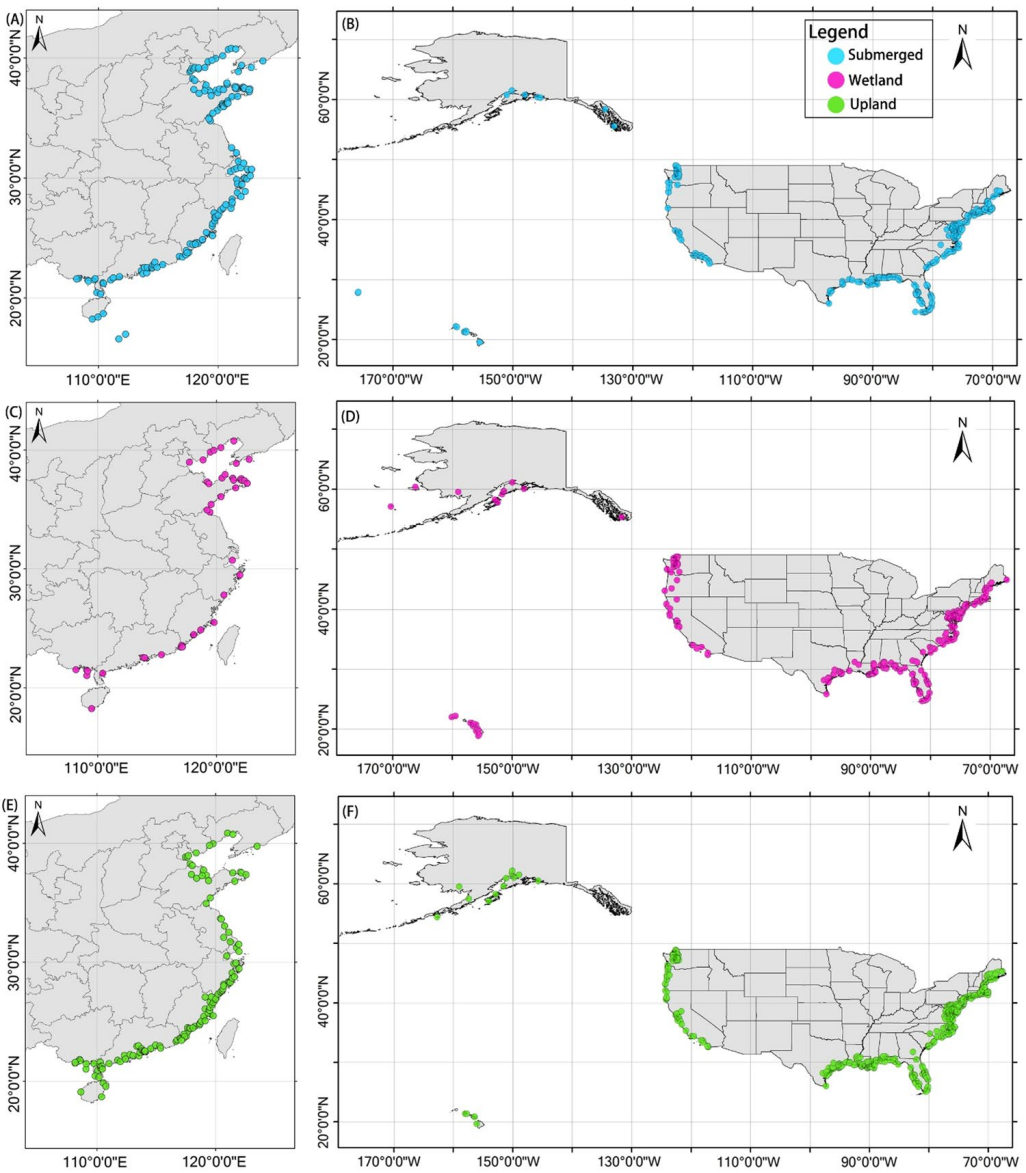
Study areas in China and the USA. (**A**) Distribution of coastal submerged areas in China. (**B**) Distribution of coastal submerged areas in the USA. (**C**) Distribution of coastal wetlands in China. (**D**) Distribution of coastal wetlands in the USA. (**E**) Distribution of coastal upland areas in China. (**F**) Distribution of coastal upland areas in the USA. (Although each of the projects is represented by a single point, many involve activities that cover large areas at multiple project locations. Refer to the project descriptions and related information to better understand the full spatial extent of the project activities and their impacts. Some newly initiated projects may not yet appear on the map).

For the period of 1992–2014, we identified 914 coastal restoration projects located in the 11 coastal provinces of China (Fig. 1, Table S1). Forty eight percent of these were wetland projects, 43% submerged projects and 9% upland projects. About 20% of the information that we collected came from government reports and web sites, and about 80% from scientific papers. For the same time period, we identified 1,620 coastal restoration projects in 23 coastal states of the USA (Fig. 1, Table S1). Fifty one percent of these were wetland projects, 28% submerged projects and 21% upland projects. Almost all of the information that we collected about projects in the USA came from National Oceanic and Atmospheric Administration (NOAA) and National Estuaries Restoration Inventory (NERI) databases.

**Figure 2:**
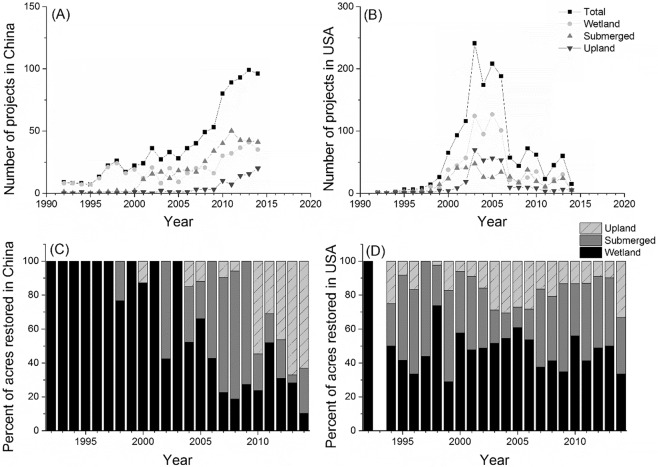
Number of new coastal restoration projects started each year from 1992 to 2014 in (**A**) China and (**B**) the USA. Projects are grouped into three broad habitat types. Restoration effort as a function of habitat type over time in (**C**) China and (**D**) the USA. Data are the percent of acres of each habitat type restored per year.

The history of coastal restoration projects is dramatically different between China and the USA (Fig. 2). In China, the overall pattern is of continual increase in new projects (Fig. 2A). The years 1992 to 1999 represented the beginning of significant restoration activity, with most of the projects focused on submerged habitats. The years 2000 to 2009 saw continued activity in restoring submerged habitats, with many new projects in wetland and upland habitats. After 2009 there was a sharp increase in the number of new projects in all three kinds of habitats. In the USA, the overall pattern is of a surge of activity between 2000 and 2006 (Fig. 2B). The years 1992 to 1999 represented a gradual ramping up of new restoration projects in all habitat types. From 2000 to 2006 there was a dramatic peak in restoration activity in all 3 habitat types. After 2006, roughly coinciding with a downturn in the USA and the global economy, there was a significant slowdown in new restoration projects.

The coastal restoration projects in the two countries were unevenly distributed across habitat types. The projects represented an accumulated 300,521 acres in China, of which 116,143 acres were wetlands (~39%), most of which were mangroves (79,637 acres) (Fig. 2C, Table 1). An additional 117,320 acres were submerged habitats (~39%), almost all of which were “coastal waters” (117,176 acres). The remaining 67,058 acres were upland (~22%), all of which were “sandy beach”. Compared to China, coastal restoration projects in the USA covered fewer acres were disproportionately conducted in wetlands. In the USA, the projects represented an accumulated 243,064 acres (Fig. 2D, Table 1), of which 162,469 acres were wetlands (~67%), most of which were salt marsh (112,196 acres), followed by freshwater marshes (11,302 acres). Only 21,502 acres were submerged habitats (~9%), most of which were ponds (14,365 acres), and the remaining 59,092 acres were upland (~24%).

**Table 1:** Different restoration techniques applied to different types of restored habitat in USA and China. A more detailed version of this table is presented in the Appendix (Table S2).

	Habitat Categories Restored	Habitat Types Restored	Restored acres	Restoration Techniques (number of total techniques)
China	Wetland	Tidal wetland	35,718	berm/dike removal, bird habitat enhancement, debris removal, fish farm removal, freshwater introduction, invasive removal, pest control, tidal channel excavation, topography reconstruction, vegetation planting, other (10).
		Mangrove	79,637	
	Submerged	Seagrass	26.96	berm/dike removal, coral larvae supplement, coral transplant, debris removal, fish farm removal, marine ranching, reef construction, stock enhancement, vegetation planting, other (9).
		Coral reef	116.38	
		Coastal waters	117,176	
	Upland	Sandy beach	67,846	beach nourishment, debris removal, fish farm removal, other (3).
USA	Wetland	Forested wetland	6,363	beach nourishment, berm/dike modification (including replacement), berm/dike removal, bird habitat enhancement, bulkhead removal, contaminant removal/remediation, culvert modification (including replacement), culvert removal, dam modification (including replacement), dam removal, debris removal, erosion control, fencing/netting, fill removal, fish exclusion devices, fish passage, invasives removal: fauna/ vegetation, land acquisition, large woody debris/structure placement, native plant nursery construction, nutrient management, placement of dredge material, planting, prescribed burn, reef construction: artificial materials, reef construction: natural materials, signage, species reintroduction (non-plant), stock enhancement, storm water/runoff controls, stream channel rehabilitation/creation, stream flow modification, stream pool construction, substrate modification, terracing, tide gate installation, tide gate modification (including replacement), tide gate removal, weir construction (39).
		Tidal wetland	5,683	
		Freshwater marsh	11,302	
		Salt marsh	112,196	
		Mangrove	5,040	
		Shrub swamp (non-mangrove)	2,027	
		Others	19,860	
	Submerged	Submerged aquatic vegetation	1,192	bird habitat enhancement, contaminant removal/remediation, coral reattachment, coral reef construction, coral stabilization, coral transplant, culvert modification (including replacement), debris removal, erosion control, fill removal, fish hatchery construction, fish passage, fishway, invasives removal: fauna/ vegetation, large woody debris/structure placement, native plant nursery construction, nutrient management, oyster gardening, oyster reef/shell bottom, planting, reef construction: artificial materials, reef construction: natural materials, signage, species reintroduction (non-plant), stock enhancement, storm water/runoff controls, stream channel rehabilitation/creation, stream flow modification, submerged aquatic vegetation, substrate modification, tide gate modification (including replacement) (~30).
		Coral reef	466	
		Pond	14,365	
		Soft bottom/mud	732	
		Oyster reef/shell bottom	1,103	
		Kelp	23	
		Water column	1,418	
		Soft bottom/sand	1,756	
		Hard bottom	62	
		Others	387	
	Upland	Maritime forest	224	beach nourishment, berm/dike modification (including replacement), bird habitat enhancement, bulkhead removal, culvert modification (including replacement), culvert removal, debris removal, erosion control, fencing/netting, fill removal, fish exclusion devices, invasives removal: fauna/vegetation, land acquisition, native plant nursery construction, oyster gardening, planting, prescribed burn, reef construction: artificial materials, signage, species reintroduction (non-plant), substrate modification, other (~21).
		Dune	2,317	
		Beach	864	
		Rocky shoreline	44	
		Others	55,642	

Restoration projects in China used fewer techniques (~1 for each project, Table 1, Fig. S1) than did projects in the USA (~2 for each project). Nearly 80% of the projects in China used only one technique, but in the USA over 45% of the projects used more than one technique (Fig. S1). For example, wetland restoration projects in China typically relied on planting, whereas projects in the USA typically used a combination of erosion control, culvert modification, and planting. Similarly, submerged restoration projects in China typically relied on debris removal, whereas projects in the USA typically used a combination of coral reef construction, fish passage, tide gate modification and debris removal. In addition, upland restoration projects in China typically relied on debris removal, whereas projects in the USA typically used a combination of bird habitat enhancement, debris removal, planting and beach nourishment.

**Figure 3:**
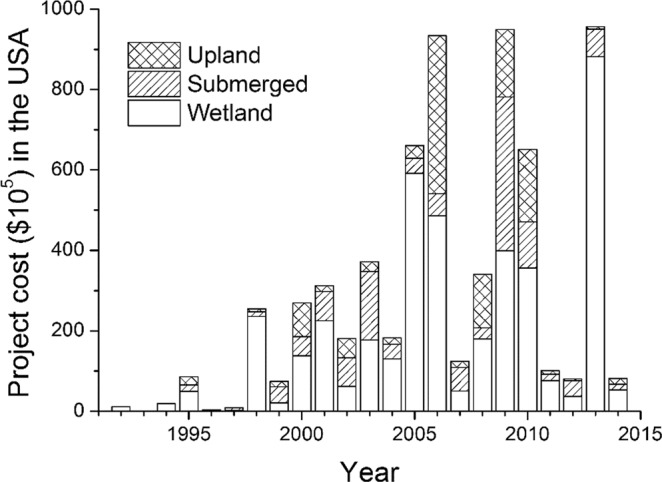
Annual costs of all new coastal restoration projects from 1992 to 2014 in the USA.

**Figure 4:**
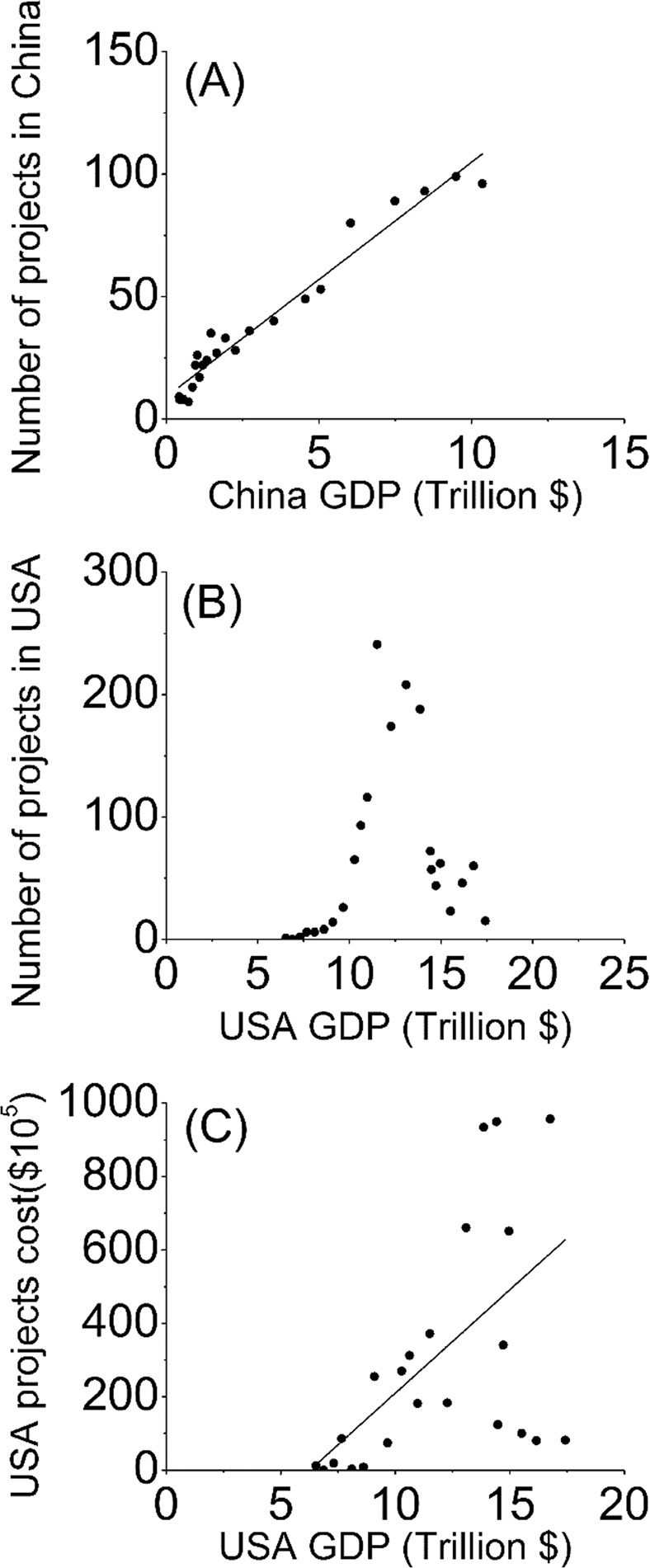
Relationships between the number of new coastal restoration projects started each year from 1992 to 2014 and the Gross domestic product (GDP) in (**A**) China (y = 9.58x + 9.05, r^2^ = 0.96, P < 0.001) and (**B**) the USA (y = 6.03x − 4.65, r^2^ = 0.04, P = 0.18), and (**C**) relationship between project costs and the GDP (y = 48.17 x −278.23, r^2^ = 0.23, P = 0.012) of the USA.

Very limited information was available on the costs of restoration projects in China (Table 2), but some information was available from online newspaper reports. For example, over 15 million US dollars have been spent on various restoration projects in the Yellow River Delta^37^, about 500 million dollars have been spent restoring mangrove forests in Futian, Guangdong province^38^, over 3 million dollars have been spent to protect and restore fish population in the Yangtze River Estuary and Hangzhou Bay^39^, and about 7 million dollars were budgeted to be spent on restoring coral reefs, mangrove forests and seagrasses in Hainan province from 2013 to 2022^40^. In the USA, over 665 million U.S. dollars have been spent on all the coastal restoration projects we studied, including over 418 million dollars (~63%) spent on wetland habitats, over 129 million dollars (~19%) on submerged habitats, and over 118 million dollars (~18%) on uplands. In the USA, there was a general increase in total project costs from 1992 to 2006, followed by a sharp decrease in 2007 and 2008, and considerable year-to-year variation thereafter (Fig. 3). The number of projects in China was positively correlated with GDP (Fig. 4A, R^2^ = 0.95, P < 0.001). The number of projects in the USA displayed a hump-shaped relationship with GDP (Fig. 4B), but total project costs had a positive relationship with GDP (Fig. 4C).

**Table 2:** Percent of coastal restoration projects in China and the USA for which each type of information was available.

Types of information available	China (%)	USA (%)
Habitat Types Restored	100	93
Restoration Techniques	97	86
Implementation start date	100	94
Implementation completion date	34	78
Longitude, Latitude	88	89
Acres	52	86
Total project cost	2	89

In China, over 98% of the projects were conducted by the government, and <2% of the projects included private parties or a non-governmental organization (NGO) as partners^31^. In contrast, in the USA, most projects were conducted by a diverse group of partners, including the government, research institutes, colleges, environmental consultants and conservation groups. Only 7% of the projects had only one partner, the average number of partners was 4, and 3% of the projects had over 10 partners. There was a striking relationship between the number of partners conducting the project and total project costs: projects with few partners could be either inexpensive or expensive, but project with many partners were always relatively expensive (Fig. S2). Because there were no inexpensive projects with many partners, there was a significant positive relationship between project cost and the number of partners (Spearman’s rho 0.27, P < 0.001).

The project implementation time period is the time it takes to construct the project, without considering post-construction monitoring. We could not find information on the project implementation time period for ~66% of the projects in China and ~22% in USA (Table 2). Based on the projects with data, projects in China took considerably longer (typically 20–70 months) to implement than projects in the USA (typically 1–30 months) (Fig. S3).

## Discussion

Coastal restoration projects vary in their specific locations, aims, scale, types and costs. Evaluating the benefits of the projects is outside the scope of this paper: it is complicated to evaluate the economic value of the multiple ecosystem services provided by restoration projects^4^, and probably for this reason, comprehensive data on benefits were not available for most projects. China and the USA have both spent hundreds of millions of dollars to implement hundreds of coastal restoration projects since 1992. Many aspects of these restoration projects, however, differ between the two countries. China and the USA differ in which types of habitats are restored, the historical pattern of restoration, the number and size of projects, the funding sources for projects, the number and type of partners involved, and in how information about projects is made available to the public.

Both China and the USA prioritized wetland restoration, implicitly recognizing the many ecosystem services provided by wetlands^4,31^. China, however, invested far less effort into upland projects than did the USA. This is likely because the large human population along the Chinese coast has appropriated a large proportion of the land for human uses^23,41^. Because coastal human populations worldwide are about three times as dense as inland populations, human pressure on coastal upland habitats is a global problem^42^. Other factors limiting the number of restoration projects in coastal upland and submerged regions may include private ownership of upland habitats and the difficulty of constructing restoration projects in submerged habitats^4^.

Restoration activity in China showed a pattern of continual increase from 1992 through 2014, but sharply dropped in the USA following a peak in 2000–2006. The drop in the number of new projects in the USA in 2007 may be explained by the economic downturn in the USA^43^ that was largely avoided in China^44^. After 2007, the number of new coastal restoration projects in the USA has remained relatively low, but funding was high in 2009, 2010 and 2013, reflecting investment in a few, larger projects such as the “Sears Point Tidal Wetland Restoration” ($18 million dollars) (http://www.sfbayjv.org/project-sears-point-wetland-restoration-san-pablo-bay.php) and the “Smith Island Estuary Restoration” ($64 million dollars) (https://restoration.atlas.noaa.gov/src/html/index.html). Some of these large projects were funded by economic stimulus money that was appropriated after the recession with the goal of rapidly injecting government spending into the economy (https://www.propublica.org/special/the-stimulus-plan-a-detailed-list-of-spending; http://www.noaanews.noaa.gov/stories2009/20090710_newport.html). The goal of rapidly spending large amounts of money naturally favored large projects. Projects may also be getting more expensive because stakeholders are favoring larger projects, or because the relatively easy, inexpensive projects (e.g., breaching dikes to restore water flow to wetlands) have largely been done.

China had fewer projects than the USA, but a similar number of restored acres. This is partially explained by a number of large projects planting mangrove forests in China that are popular in part because they are easy to conduct and inexpensive^4^. Many of these planting projects, however, used non-native mangrove species, making it debatable whether they should be considered as true restoration projects or instead as forestry plantations^45–47^.The traits of the non-native mangrove species that have commonly been used in these projects, such as fast growth, high salt tolerance, great reproductive capacity, and rapid alteration of habitat structure, made them suitable rapidly growing forests and protecting coastlines. At the same time, the rapid expansion of non-native mangrove species on the Chinese coastline threatens native species and ecosystems. We concur with calls for future mangrove restoration projects to transition to use of native species as soon as possible so as to achieve not just the establishment of forest cover, but true ecosystem restoration^45^.

In general, conservation biologists encourage habitat protection or restoration on a large scale^48,49^. Large projects should produce more intact ecosystems that are both more likely to recover successfully and to support more ecosystem services than small scale projects^49,50^. However, many factors like funding or regional policies can limit project scale. In addition, small-scale projects might leverage local expert knowledge and can be a primary test for restoration techniques. Size alone does not guarantee success: if a large scale project has unclear objectives or is poorly coordinated, it will result in poor outcomes^50^. Therefore, both small and large projects can be valuable under different circumstances^48,51,52^. Moreover, a network of small protected areas may create connectivity at a regional scale, bringing benefits that a single large project of the same total acreage would not^53–55^. For example, the state of Louisiana began restoring the Mississippi delta in the 1930s, and currently is implementing a $50 billion, 50-year coastal master plan (revised at 5-year intervals) to reduce flood risk for developed areas and restore prioritized deltaic wetlands to a more self-sustaining and healthy condition. Because of the scale of this project, it is able to comprehensively address 21^st^ century global change megatrends including climate change, energy scarcity and ecosystem degradation, and to do so at a landscape scale while considering economic constraints and the local cultural context^56^.

Many kinds of restoration techniques have been used in China and the USA (Table 1, Fig. S1). The majority of projects in China used only a single restoration technique. In contrast, more than half of the projects in USA used multiple techniques. This difference may reflect a greater complexity of projects in the USA that requires multiple techniques—for example, projects in China, restoring wetlands may just require planting if the problem was just that the mangroves had been cut and needed to be replanted. In the US, restoring wetlands typically involves creating new intertidal habitat from upland, so it requires both excavating and planting. More techniques do not necessarily imply a more successful outcome. We did not evaluate how successful the different approaches were, but in general restoration practice has developed more as an art than as a science. Scientists frequently call for restoration projects to include experiments to compare techniques, but projects are rarely done in this way. Because restoration projects are usually not done as experiments, and in fact are often evaluated only superficially, it is likely that the state of art in both countries falls far short of what could be achieved for the same amount of funding^4,57,58^.

Restoration projects require funding. The increasing number of new restoration projects and the implementation of larger projects both reflect increased funding^4,59,60^. Funding for restoration projects in China has benefitted from the steady increase in national GDP. In contrast, funding for new projects in the USA appears to have suffered during the economic downturn and fluctuated thereafter. Inadequate funding for restoration tends to promote smaller, poorly-planned projects that are inadequately monitored, and may even cause some projects to be abandoned in the middle of implementation^61^. In China, funding for restoration comes almost entirely from the national government, which to date has led to a relatively stable funding situation. In the USA, funding for restoration comes from both federal and private sources, and some restoration is conducted as mitigation for development projects. During an economic downturn, tax revenues decline, private donations decrease, and development that might require mitigation also decreases, all of which may lead to restoration activity varying over time. If the government conducts restoration projects as part of an economic stimulus, however, spending on restoration can increase during a depression, as we saw in the USA in 2009.

Projects in China had fewer partners than projects in the USA. In China, most of the coastal restoration projects are sponsored, organized and implemented by the government. In the USA, government and non-governmental organizations sponsored and implemented projects together. Having a central role for the government can produce several advantages. The government is able to fund large-scale projects, has the resources to manage large projects, and can enforce consistent methods for implementing and evaluating projects^62^. On the other hand, although having multiple partners may reduce efficiency and create additional challenges for project management^63,64^, it may also increase local knowledge and the breadth of interdisciplinary skills available^31^. Moreover, in the USA, complicated patterns of property ownership and legal responsibility often require that multiple partners are engaged in projects. Projects in China took longer to implement than in the USA, probably because they did not have the same economic urgency. In particular, developers doing restoration as mitigation in the USA have a strong financial incentive to complete projects quickly. We did not evaluate how long projects were monitored, but other reviews have pointed out that monitoring of restoration projects in the USA is typically too short to adequately assess whether the restored habitats are fully developed or functional^58,65^. It is likely that the same concern applies in China. We also did not evaluate whether projects were self-sustaining or required continued human management in order to function. The former is less expensive, but continued management may be necessary if habitats are degraded, invaded by exotic species, or lack historic natural processes such as fires or floods.

We were able to gather a large amount of information about coastal restoration projects in both countries by searching online; however, in the USA this information is systematically organized by government agencies in a small number of websites, whereas in China we obtained most of our information from scientific papers. China would benefit from better documenting and systematically presenting information about restoration projects. Moreover, our database of projects in both countries was incomplete, with a high proportion of missing entries, especially in China (Table 2). In addition, China could take lessons from the Northwest Fisheries Science Center, 2019: Coastal Assessment Framework - National Assessment of Estuary and Coastal Habitats (https://inport.nmfs.noaa.gov/inport/item/30858), that is to build a construction file for each project, including item identification, physical location, data set information, support roles, extents, access information, distribution information, data quality, data management, lineage, child items and catalog details. Thus, our understanding of the history of restoration activity in both countries was hindered by incomplete and inconsistent availability of records.

## Conclusion

In summary, China and the USA both spent hundreds of millions of dollars to implement hundreds of coastal restoration projects during 1992–2014. Many aspects of these projects differed between the two countries, including the types of habitats restored, the historical pattern of restoration, the number and size of projects, the funding sources for projects, the number and type of partners involved, and how information about projects was made available to the public. Both countries, but especially China, could do more to make information about restoration projects publically available. For both countries, we know more about project construction than about project outcomes, making it unclear whether projects are achieving their goals in a cost-effective manner. We recommend that China develop an online database of restoration projects similar to those available in the USA, that both countries work to ensure that project documentation in these databases is complete, and that both countries expand the databases to include information about project outcomes based on long-term monitoring.

In China, rapid population growth along the coast has produced enormous pressure on upland habitats, and very little funding has gone to restoring these habitats. China might benefit by doing more restoration in coastal upland habitats, especially when projects can be done adjacent to wetlands so that species can move back and forth between upland and wetland habitats^23,31^.

## Methods

Coastal habitats, in a narrow sense, are habitats that are periodically flooded by seawater, namely rocky intertidal, the intertidal portion of sandy beaches, and coastal wetlands (variously known as salt marsh, mangrove, freshwater marsh, forested wetland, tidal wetland, riparian zone, shrub swamp, etc.). However, in a broad sense, they also include the supra-tidal (henceforth upland, including habitats categorized as beach, sandy beach, maritime forest, rocky shoreline, dunes, etc.) and sub-tidal (henceforth submerged, including habitats categorized as submerged aquatic vegetation, coral reef, pond, hard bottom, soft bottom, mud/sand, oyster reef/shell bottom, kelp forest, riverine, in-stream, water column, etc.) areas (Table 1). We used the broader definition in this paper.

To compare coastal restoration projects between China and the USA, we collected information from two online publically-accessible resources (the National Oceanic and Atmospheric Administration (NOAA) Habitat Restoration Projects database at https://restoration.atlas.noaa.gov/src/html/index.html and the National Estuaries Restoration Inventory at NERI, https://neri.noaa.gov/neri/home.html), the primary literature, government reports and online news. To review the primary literature, we searched Web of Science (http://apps.webofknowledge.com) for papers published in English, and the China Knowledge Resource Integrated Database (CNKI, http://www.cnki.net) for papers published in Chinese. We searched for (restor * OR recover OR rehab* OR creat*) AND (tidal OR marsh OR saltmarsh OR intertidal OR coast* OR estuary OR delta OR beach OR mangrove OR coral OR oyster OR seagrass OR nearshore OR shoreline), and used similar search terms in Chinese^4,31^. Since coastal restoration projects reported by NOAA in the USA started in 1992, we collected information about projects in China that started in the same year. We ended with projects that started in 2014. For each project, we recorded what type of habitat was restored, how large the project was, the date the project was started, the date that it was completed, the cost of the project (the Chinese RMB was converted to US dollars at a rate of 6 RMB = 1 dollar; expenses were not adjusted for inflation), how many organizations partnered to conduct the project, and what techniques were employed in the restoration. Not all this information was available for each project. Coastal habitats were broadly categorized as upland, submerged, riverine and wetlands^66^. Since different types of habitat support different ecosystem services, we further categorized habitats following the National Estuaries Restoration Inventory. Freshwater habitats were included if they fell within the coastal zone. For unclear or lost types of habitat information, we used “other” as a category. Information about project outcomes was not consistently available, and so we did not attempt to evaluate project success or cost-effectiveness.

We also collected data on the gross domestic product (GDP) of China and the USA from the World Bank website (http://www.worldbank.org/) from 1992 to 2014. GDP was reported in current U.S. dollars and was not adjusted for inflation. We related the number of new projects started each year in China and in the USA to GDP across 23 years using linear regression. We also correlated total project cost to the number of partners in USA to test the hypothesis that projects with more partners would cost more.

The statistical analyses were conducted using SPSS 20 software (International Business Machine Corporation, Armonk, New York, USA) and Origin 8.5 (OriginLab Corporation, Northampton, MA, USA).

## Supplementary information

Supplement information: A comparison of coastal habitat restoration projects in China and the United States
