# Supplementary material for: A comparison of coastal habitat restoration projects in China and the United States
Source: Sci Rep. 2019 Oct 7;9:14388. doi: 10.1038/s41598-019-50930-6 (PMC6779881; doi:10.1038/s41598-019-50930-6)
Supplement: Supplementary file 1 — Supplement information: A comparison of coastal habitat restoration projects in China and the United States [file 41598_2019_50930_MOESM1_ESM.doc]

**Supplement information**

**A comparison of coastal habitat restoration projects in China and the United States**

**Authors**: Shanze Li^1,2^, Tian Xie^3^, Steven C. Pennings^4^, Yuchun Wang^1,2*^, Christopher Craft^5^, Mingming Hu^1,2^

**Affiliations**: ^1^ State Key Laboratory of Simulation and Regulation of Water Cycle in River Basin, China Institute of Water Resources and Hydropower Research, Beijing, 100038, China

^2^ Department of Water Environment, China Institute of Water Resources and Hydropower Research, Beijing, 100038, China

^3^ School of Environment, Beijing Normal University, State Key Joint Laboratory of Environmental Simulation and Pollution Control, Beijing 100875, China

^4^ Department of Biology and Biochemistry, University of Houston, Houston TX 77204, USA.

^5^ School of Public and Environmental Affairs, Indiana University, Bloomington, IN 47405, USA.

***Correspondence authors:** Yuchun Wang, E-mail: wangyc@iwhr.com, Tel: +86 10 68781881

**Author Contributions:** SL, TX, and SCP designed the study; SL and TX analyzed the data; SL, TX and SCP wrote the manuscript; CC, YW, MH helped improve the Manuscript, and all authors contributed substantially to revisions.

**Competing interests:**

The authors declare no competing interests.

**Acknowledgements**

This study was supported financially by National Science Foundation for Young Scientists of China (51809287), China Postdoctoral Science Foundation Grant (2018M630182, 212400248, 2018M641249), and was funded by the Open Research Fund of State Key Laboratory of Simulation and Regulation of Water Cycle in River Basin, China Institute of Water Resources and Hydropower Research (SKL2018ZY04). SCP and CC were supported by the US National Science Foundation through the Georgia Coastal Ecosystems Long-Term Ecological Research program under Grant No. OCE-1237140.

Table S1 Coastline length and number of coastal restoration projects in different provinces in China and different states in the USA.

|  | China |  |  | USA |  |  |
| --- | --- | --- | --- | --- | --- | --- |
| No. | Province | Coastline (km) | Number of projects | State | Coastline (km) | Number of projects |
| 1 | Fujian | 3023.6 | 164 | Alabama | 53 | 30 |
| 2 | Guangdong | 4314.1 | 200 | Alaska | 6,640 | 35 |
| 3 | Guangxi | 1478.2 | 40 | California | 840 | 111 |
| 4 | Hainan | 235 | 36 | Connecticut | -- | 21 |
| 5 | Hebei | 487.3 | 44 | Delaware | 28 | 94 |
| 6 | Jiangsu | 1039.7 | 33 | Florida | 1,350 | 251 |
| 7 | Liaoning | 2178.3 | 51 | Georgia | 100 | 9 |
| 8 | Shandong | 3124.4 | 179 | Hawaii | 750 | 58 |
| 9 | Shanghai | 167.8 | 40 | Louisiana | 397 | 95 |
| 10 | Tianjin | 133.4 | 10 | Maine | 228 | 50 |
| 11 | Zhejiang | 2253.7 | 107 | Maryland | 31 | 134 |
| 12 | China |  | 10 | Massachusetts | 192 | 88 |
| 13 |  |  |  | Mississippi | 44 | 18 |
| 14 |  |  |  | New Hampshire | 13 | 15 |
| 15 |  |  |  | New Jersey | 130 | 83 |
| 16 |  |  |  | New York | 127 | 76 |
| 17 |  |  |  | North Carolina | 301 | 78 |
| 18 |  |  |  | Oregon | 296 | 40 |
| 19 |  |  |  | Rhode Island | 40 | 42 |
| 20 |  |  |  | South Carolina | 187 | 31 |
| 21 |  |  |  | Texas | 367 | 76 |
| 22 |  |  |  | Virginia | 112 | 62 |
| 23 |  |  |  | Washington | 157 | 123 |
| Total |  | 18436 | 914 |  | 12383 | 1620 |

Table S2 Different restoration techniques applied to different types of restored habitat in USA and China. This is an expanded version of Table 1 in the manuscript.

| **Country** | **Habitat Categories Restored** | **Habitat Types Restored** | **Restored acres** | **Restoration Techniques** |
| --- | --- | --- | --- | --- |
| China | wetland | Tidal marsh | 35,718 | Berm/dike removal |
| China | wetland | Tidal marsh |  | Bird habitat enhancement |
| China | wetland | Tidal marsh |  | Debris removal |
| China | wetland | Tidal marsh |  | Dredging |
| China | wetland | Tidal marsh |  | Fish farm removal; |
| China | wetland | Tidal marsh |  | Freshwater introduction; |
| China | wetland | Tidal marsh |  | Invasives removal |
| China | wetland | Tidal marsh |  | Pest control |
| China | wetland | Tidal marsh |  | Stock enhancement |
| China | wetland | Tidal marsh |  | Tidal channel excavation |
| China | wetland | Tidal marsh |  | Topography reconstruction |
| China | wetland | Tidal marsh |  | Vegetation planting |
| China | wetland | Mangrove | 79,637 | Debris removal |
| China | wetland | Mangrove |  | Dredging |
| China | wetland | Mangrove |  | Fish farm removal |
| China | wetland | Mangrove |  | Freshwater introduction |
| China | wetland | Mangrove |  | Invasives removal |
| China | wetland | Mangrove |  | Vegetation planting |
| China | Submerged | Seagrass | 26.96 | Fish farm removal |
| China | Submerged | Seagrass |  | Vegetation planting |
| China | Submerged | Coral reef | 116.38 | Coral larvae supplement |
| China | Submerged | Coral reef |  | Coral transplant |
| China | Submerged | Coral reef |  | Reef construction |
| China | Submerged | Coastal waters | 117,176 | Reef construction |
| China | Submerged | Coastal waters |  | Berm/dike removal |
| China | Submerged | Coastal waters |  | Debris removal |
| China | Submerged | Coastal waters |  | Dredging |
| China | Submerged | Coastal waters |  | Fish farm removal |
| China | Submerged | Coastal waters |  | Marine ranching |
| China | Submerged | Coastal waters |  | Reef construction |
| China | Submerged | Coastal waters |  | Stock enhancement |
| China | Submerged | Coastal waters |  | Vegetation planting |
| China | upland | Sandy beach | 67,846 | Beach nourishment |
| China | upland | Sandy beach |  | Debris removal |
| China | upland | Sandy beach |  | Dredging |
| China | upland | Sandy beach |  | Fish farm removal |
| China | upland | Sandy beach |  | Freshwater introduction |
| USA | wetland | forested wetland | 6,363 | berm/dike modification (including replacement) |
| USA | wetland | forested wetland |  | invasives removal: fauna |
| USA | wetland | forested wetland |  | invasives removal: vegetation |
| USA | wetland | forested wetland |  | large woody debris/structure placement |
| USA | wetland | forested wetland |  | nutrient management |
| USA | wetland | forested wetland |  | planting |
| USA | wetland | forested wetland |  | storm water/runoff controls |
| USA | wetland | forested wetland |  | stream channel rehabilitation/creation |
| USA | wetland | Tidal wetland | 5,683 | planting |
| USA | wetland | freshwater marsh | 11,302 | berm/dike modification (including replacement) |
| USA | wetland | freshwater marsh |  | berm/dike removal |
| USA | wetland | freshwater marsh |  | bird habitat enhancement |
| USA | wetland | freshwater marsh |  | culvert modification (including replacement) |
| USA | wetland | freshwater marsh |  | dam modification (including replacement) |
| USA | wetland | freshwater marsh |  | dam removal |
| USA | wetland | freshwater marsh |  | debris removal |
| USA | wetland | freshwater marsh |  | erosion control |
| USA | wetland | freshwater marsh |  | fencing/netting |
| USA | wetland | freshwater marsh |  | fill removal |
| USA | wetland | freshwater marsh |  | fish passage |
| USA | wetland | freshwater marsh |  | invasives removal: fauna |
| USA | wetland | freshwater marsh |  | invasives removal: vegetation |
| USA | wetland | freshwater marsh |  | large woody debris/structure placement |
| USA | wetland | freshwater marsh |  | planting |
| USA | wetland | freshwater marsh |  | stock enhancement |
| USA | wetland | freshwater marsh |  | stream channel rehabilitation/creation |
| USA | wetland | freshwater marsh |  | stream flow modification |
| USA | wetland | freshwater marsh |  | stream pool construction |
| USA | wetland | freshwater marsh |  | terracing |
| USA | wetland | freshwater marsh |  | tide gate installation |
| USA | wetland | freshwater marsh |  | tide gate modification (including replacement) |
| USA | wetland | freshwater marsh |  | tide gate removal |
| USA | wetland | freshwater marsh |  | weir construction |
| USA | wetland | salt marsh | 112,196 | beach nourishment |
| USA | wetland | salt marsh |  | berm/dike modification (including replacement) |
| USA | wetland | salt marsh |  | berm/dike removal |
| USA | wetland | salt marsh |  | bird habitat enhancement |
| USA | wetland | salt marsh |  | bulkhead removal |
| USA | wetland | salt marsh |  | contaminant removal/remediation |
| USA | wetland | salt marsh |  | culvert modification (including replacement) |
| USA | wetland | salt marsh |  | culvert removal |
| USA | wetland | salt marsh |  | dam removal |
| USA | wetland | salt marsh |  | debris removal |
| USA | wetland | salt marsh |  | erosion control |
| USA | wetland | salt marsh |  | fencing/netting |
| USA | wetland | salt marsh |  | fill removal |
| USA | wetland | salt marsh |  | fish exclusion devices |
| USA | wetland | salt marsh |  | fish passage |
| USA | wetland | salt marsh |  | invasives removal: fauna |
| USA | wetland | salt marsh |  | invasives removal: vegetation |
| USA | wetland | salt marsh |  | large woody debris/structure placement |
| USA | wetland | salt marsh |  | native plant nursery construction |
| USA | wetland | salt marsh |  | placement of dredge material |
| USA | wetland | salt marsh |  | planting |
| USA | wetland | salt marsh |  | prescribed burn |
| USA | wetland | salt marsh |  | signage |
| USA | wetland | salt marsh |  | stream channel rehabilitation/creation |
| USA | wetland | salt marsh |  | stream flow modification |
| USA | wetland | salt marsh |  | substrate modification |
| USA | wetland | salt marsh |  | terracing |
| USA | wetland | salt marsh |  | tide gate installation |
| USA | wetland | salt marsh |  | tide gate modification (including replacement) |
| USA | wetland | salt marsh |  | weir construction |
| USA | wetland | mangrove | 5,040 | berm/dike removal |
| USA | wetland | mangrove |  | contaminant removal/remediation |
| USA | wetland | mangrove |  | culvert modification (including replacement) |
| USA | wetland | mangrove |  | debris removal |
| USA | wetland | mangrove |  | erosion control |
| USA | wetland | mangrove |  | fill removal |
| USA | wetland | mangrove |  | invasives removal: fauna |
| USA | wetland | mangrove |  | invasives removal: vegetation |
| USA | wetland | mangrove |  | native plant nursery construction |
| USA | wetland | mangrove |  | planting |
| USA | wetland | mangrove |  | stream channel rehabilitation/creation |
| USA | wetland | mangrove |  | substrate modification |
| USA | wetland | mangrove |  | tide gate modification (including replacement) |
| USA | wetland | Shrub swamp (non-mangrove) | 2,027 | invasives removal: fauna |
| USA | wetland | Shrub swamp (non-mangrove) |  | invasives removal: vegetation |
| USA | wetland | others | 19,860 |  |
| USA | Submerged | submerged aquatic vegetation | 1,192 | debris removal |
| USA | submerged | submerged aquatic vegetation |  | fish passage |
| USA | submerged | submerged aquatic vegetation |  | invasives removal: fauna |
| USA | Submerged | submerged aquatic vegetation |  | invasives removal: vegetation |
| USA | Submerged | submerged aquatic vegetation |  | nutrient management |
| USA | submerged | submerged aquatic vegetation |  | planting, |
| USA | submerged | submerged aquatic vegetation |  | reef construction: artificial materials |
| USA | submerged | submerged aquatic vegetation |  | signage |
| USA | submerged | submerged aquatic vegetation |  | stock enhancement |
| USA | Submerged | submerged aquatic vegetation |  | storm water/runoff controls |
| USA | submerged | submerged aquatic vegetation |  | stream channel rehabilitation/creation |
| USA | submerged | submerged aquatic vegetation |  | stream flow modification |
| USA | submerged | submerged aquatic vegetation |  | substrate modification |
| USA | Submerged | Coral reef | 466 | coral reattachment |
| USA | Submerged | Coral reef |  | coral reef construction |
| USA | Submerged | Coral reef |  | coral stabilization |
| USA | Submerged | Coral reef |  | coral transplant |
| USA | Submerged | Coral reef |  | debris removal |
| USA | Submerged | Coral reef |  | invasives removal: fauna |
| USA | Submerged | Coral reef |  | invasives removal: vegetation |
| USA | Submerged | Coral reef |  | planting |
| USA | Submerged | Coral reef |  | stock enhancement |
| USA | Submerged | Pond | 14,365 | bird habitat enhancement |
| USA | Submerged | Pond |  | culvert modification (including replacement) |
| USA | Submerged | Pond |  | fish passage |
| USA | Submerged | Pond |  | fishway |
| USA | Submerged | Pond |  | invasives removal: vegetation |
| USA | Submerged | Pond |  | large woody debris/structure placement |
| USA | Submerged | Pond |  | native plant nursery construction |
| USA | Submerged | Pond |  | planting |
| USA | Submerged | Pond |  | stream channel rehabilitation/creation |
| USA | Submerged | Pond |  | substrate modification |
| USA | Submerged | Pond |  | tide gate modification (including replacement) |
| USA | submerged | soft bottom/mud | 732 | culvert modification (including replacement) |
| USA | submerged | soft bottom/mud |  | debris removal, signage |
| USA | Submerged | Soft bottom/mud |  | stock enhancement |
| USA | Submerged | Soft bottom/mud |  | stream flow modification |
| USA | Submerged | Oyster reef/shell bottom | 1,103 | contaminant removal/remediation |
| USA | Submerged | Oyster reef/shell bottom |  | debris removal |
| USA | Submerged | Oyster reef/shell bottom |  | erosion control |
| USA | Submerged | Oyster reef/shell bottom |  | fish hatchery construction |
| USA | Submerged | Oyster reef/shell bottom |  | oyster gardening |
| USA | Submerged | Oyster reef/shell bottom |  | planting |
| USA | Submerged | Oyster reef/shell bottom |  | reef construction: artificial materials |
| USA | Submerged | Oyster reef/shell bottom |  | reef construction: natural materials |
| USA | Submerged | Oyster reef/shell bottom |  | species reintroduction (non-plant) |
| USA | Submerged | Oyster reef/shell bottom |  | stock enhancement |
| USA | Submerged | Oyster reef/shell bottom |  | storm water/runoff controls |
| USA | Submerged | kelp | 23 | invasives removal: fauna |
| USA | Submerged | kelp |  | planting |
| USA | Submerged | kelp |  | stock enhancement |
| USA | Submerged | Water column | 1,418 | culvert modification (including replacement) |
| USA | Submerged | Water column |  | debris removal |
| USA | Submerged | soft bottom/sand | 1,756 | contaminant removal/remediation |
| USA | Submerged | soft bottom/sand |  | debris removal |
| USA | Submerged | soft bottom/sand |  | stock enhancement |
| USA | Submerged | Hard bottom | 62 | debris removal |
| USA | Submerged | Hard bottom |  | fill removal |
| USA | Submerged | Hard bottom |  | planting |
| USA | Submerged | Hard bottom |  | reef construction: artificial materials |
| USA | Submerged | Hard bottom |  | reef construction: natural materials |
| USA | Submerged | Hard bottom |  | stock enhancement |
| USA | Submerged | Hard bottom |  | stream channel rehabilitation/creation |
| USA | Submerged | in-stream |  | culvert modification (including replacement) |
| USA | Submerged | in-stream |  | culvert removal |
| USA | Submerged | in-stream |  | dam removal |
| USA | Submerged | in-stream |  | fill removal |
| USA | Submerged | in-stream |  | fish passage |
| USA | Submerged | in-stream |  | fishway |
| USA | Submerged | in-stream |  | placement of dredge material |
| USA | Submerged | in-stream |  | planting |
| USA | Submerged | in-stream |  | stream channel rehabilitation/creation |
| USA | Submerged | in-stream |  | weir construction |
| USA | submerged | riparian zone (non-wetland) | - | invasives removal: vegetation |
| USA | Submerged | riverine | - | fish passage |
| USA | Submerged | riverine | - | fishway |
| USA | Submerged | other | 387 |  |
| USA | upland | maritime forest | 224 | invasives removal: fauna |
| USA | upland | maritime forest |  | invasives removal: vegetation |
| USA | upland | maritime forest |  | native plant nursery construction |
| USA | upland | maritime forest |  | planting |
| USA | upland | maritime forest |  | species reintroduction (non-plant) |
| USA | upland | dune | 2317 | beach nourishment |
| USA | upland | dune |  | culvert modification (including replacement) |
| USA | upland | dune |  | culvert removal |
| USA | upland | dune |  | debris removal |
| USA | upland | dune |  | erosion control |
| USA | upland | dune |  | fencing/netting |
| USA | upland | dune |  | invasives removal: vegetation |
| USA | upland | dune |  | planting |
| USA | upland | dune |  | signage |
| USA | upland | beach | 864 | bird habitat enhancement |
| USA | upland | beach |  | bulkhead removal |
| USA | upland | beach |  | debris removal |
| USA | upland | beach |  | invasives removal:vegetation |
| USA | upland | beach |  | land acquisition |
| USA | upland | beach |  | planting |
| USA | upland | beach |  | signage |
| USA | upland | rocky shoreline | 44 | debris removal |
| USA | upland | other | 55642 | berm/dike modification (including replacement) |
| USA | upland | other |  | bird habitat enhancement |
| USA | upland | other |  | erosion control |
| USA | upland | other |  | fencing/netting |
| USA | upland | other |  | fill removal |
| USA | upland | other |  | fish exclusion devices |
| USA | upland | other |  | fish passage |
| USA | upland | other |  | invasives removal: fauna |
| USA | upland | other |  | invasives removal: vegetation |
| USA | upland | other |  | land acquisition |
| USA | upland | other |  | native plant nursery construction |
| USA | upland | other |  | planting |
| USA | upland | other |  | prescribed burn |
| USA | upland | other |  | species reintroduction (non-plant) |
| USA | upland | other |  | storm water/runoff controls |
| USA | upland | other |  | substrate modification |


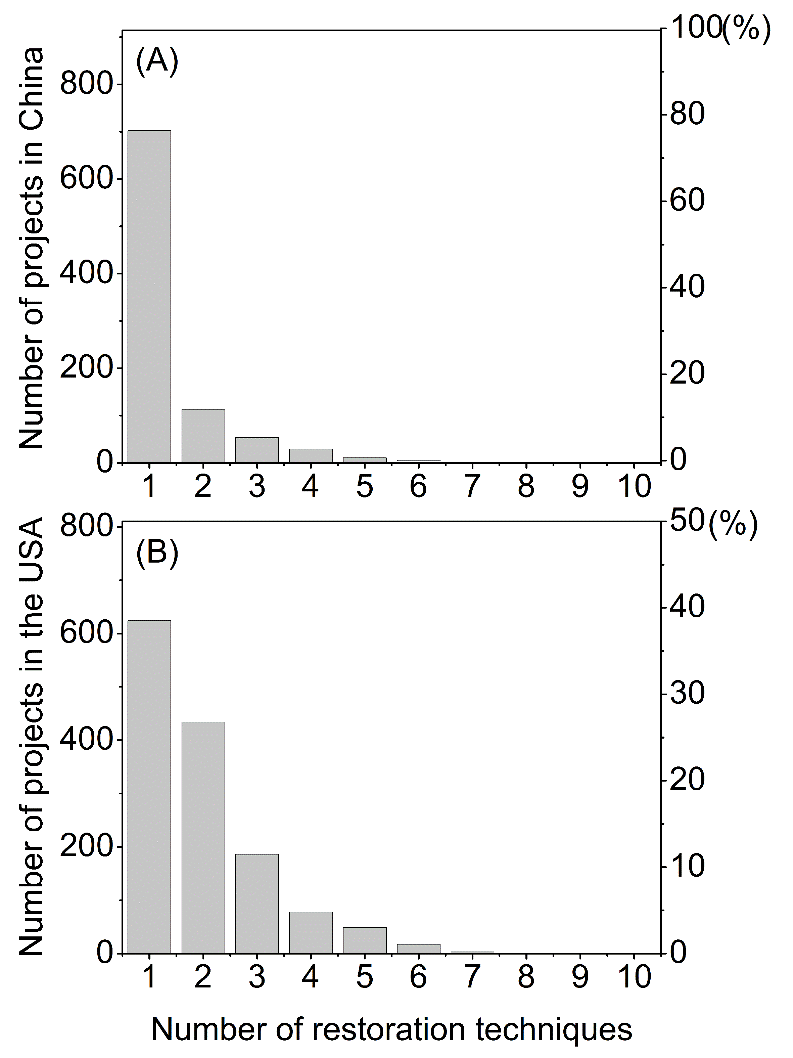


Figure S1 Frequency of projects with different numbers of restoration techniques in (A) China and (B) the USA.


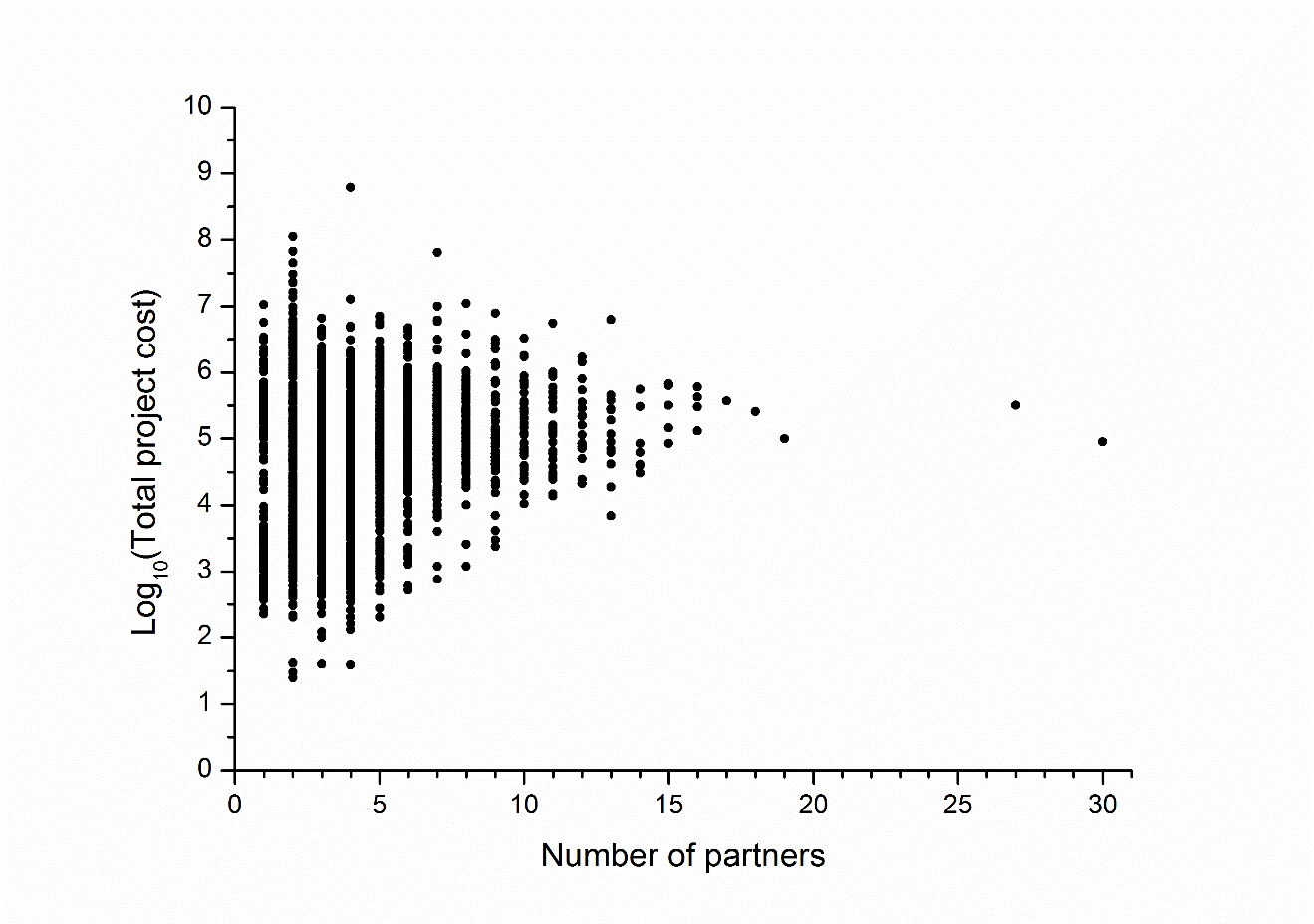


Figure S2 Relationship between total project cost (US dollars) and number of partners in the USA (Spearman’s rho 0.27, P<0.001).


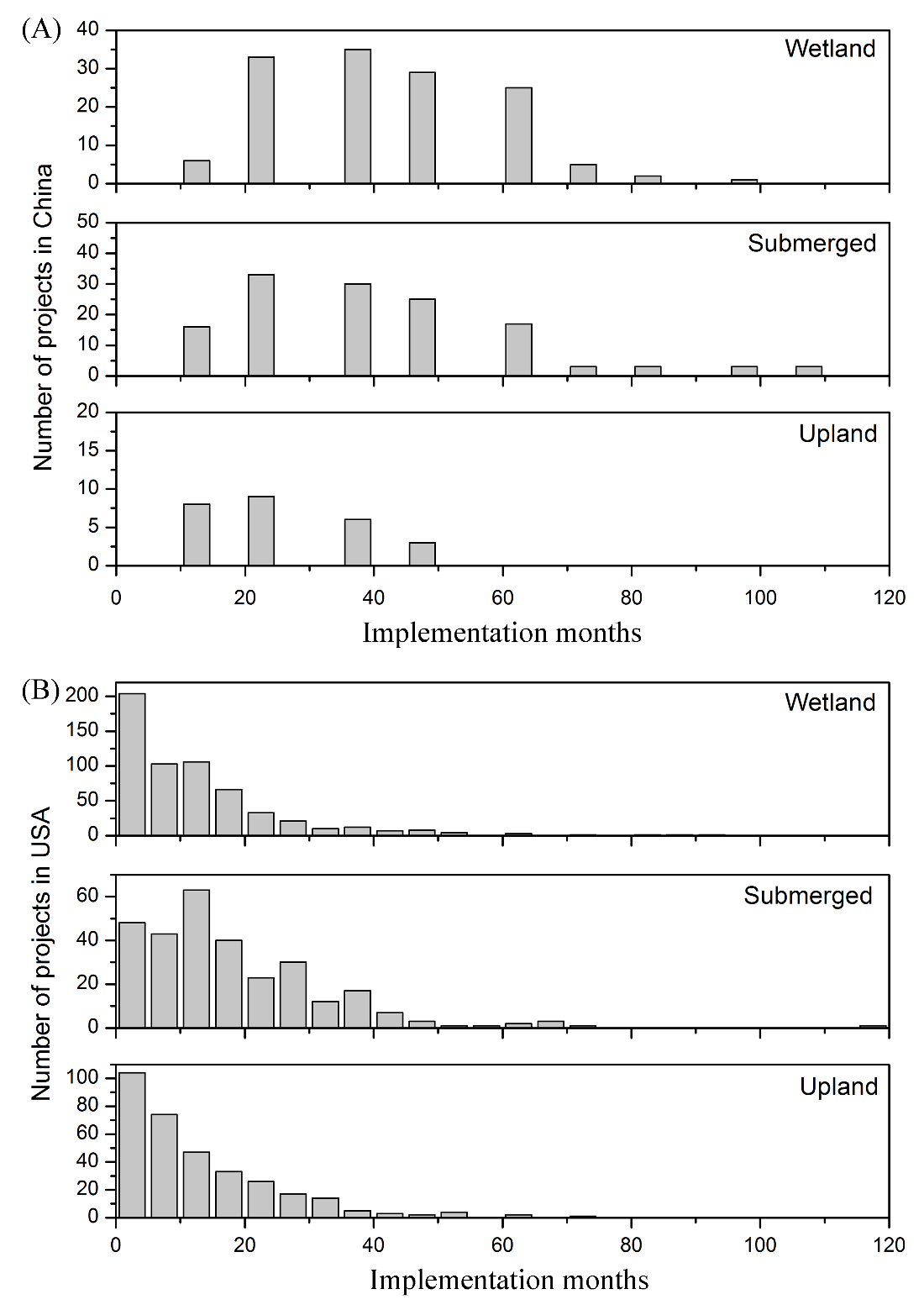


Figure S3 How long it took to implement coastal restoration projects in (A) China and (B) the USA.
